# Advancing electroencephalography education in anesthesiology

**DOI:** 10.1097/ACO.0000000000001521

**Published:** 2025-05-26

**Authors:** Joana Berger-Estilita, Sarah Saxena, Mia Gisselbaek

**Affiliations:** a Institute for Medical Education, University of Bern; b Institute of Anaesthesia and Intensive Care, Salemspital, Hirslanden Medical Group, Bern, Switzerland; c RISE-Health, Centre for Health Technology and Services Research, Faculty of Medicine, University of Porto, Porto, Portugal; d Department of Anesthesiology, Helora; e Department of Surgery, UMons, Research Institute for Health Sciences and Technology, University of Mons, Mons, Belgium; f Division of Anesthesiology, Department of Anesthesiology, Clinical Pharmacology, Intensive Care and Emergency Medicine, Geneva University Hospitals and Faculty of Medicine; g Unit of Development and Research in Medical Education (UDREM), Faculty of Medicine, University of Geneva, Geneva, Switzerland

**Keywords:** anesthesia education, competency-based training, deliberate practice, electroencephalography monitoring, perioperative brain health

## Abstract

**Purpose of review:**

Electroencephalography (EEG) monitoring is a powerful tool for optimizing anesthesia depth and improving perioperative neurocognitive outcomes. Despite its potential, EEG adoption remains limited in anesthesiology due to training gaps, perceived complexity, and reliance on processed EEG indices. This review examines current educational strategies and highlights the need for structured EEG education in anesthesia training.

**Recent findings:**

Emerging educational methodologies include short, focused teaching sessions, electronic learning modules, simulation-based practice, real-time clinical application with feedback, and self-directed study resources. Deliberate practice, spaced repetition, and competency-based progression have shown significant promise in improving EEG interpretation skills among anesthesiologists. Research remains sparse, but existing evidence indicates that even brief, structured interventions can meaningfully enhance clinical proficiency.

**Summary:**

Structured, accessible EEG training programs are critical to demystifying EEG interpretation and integrating its use into standard anesthetic practice. Embedding EEG education into residency curricula by relying on modern educational techniques and promoting faculty development will be essential to ensure anesthesiologists are equipped to use EEG monitoring effectively, ultimately improving patient outcomes.

**KEY POINTS**

- Electroencephalography (EEG) is essential for modern anesthesia practice but underutilized: despite strong physiological rationale and technological advances, EEG monitoring remains underused in anesthesiology due to limited training, perceived complexity, and cultural resistance.
- Targeted EEG education dramatically improves competence: short, structured teaching sessions (such as focused didactics, flipped classrooms, and simulation) can meaningfully enhance anesthesiologists’ ability to interpret raw EEG, debunking the myth that EEG interpretation is inherently too complex.
- Emerging educational methods show promise: modern EEG teaching integrates deliberate practice, real-time clinical application, e-learning, and simulation-based education, promoting active learning, spaced repetition, and progressive skill development aligned with competency-based medical education.
- Self-directed learning is a critical adjunct: accessible, high-quality self-study resources – including video libraries, e-learning platforms, and case-based exercises – empower learners to consolidate EEG skills independently, supporting lifelong professional growth.
- A structured, tiered curriculum is needed for sustainable integration: formal EEG education should follow a tiered, spiral model, with repeated reinforcement of basic to advanced concepts, embedded into anesthesiology training milestones and supported by faculty development to ensure the widespread, competent adoption of EEG monitoring in clinical practice.

## INTRODUCTION

Electroencephalography (EEG) in the context of anesthesiology is a monitoring technique that enables anesthesiologists to assess and manage the depth of anesthesia, ensuring patient safety during surgical procedures. First introduced in clinical practice in the late 1930s [1], EEG serves today as a sensitive tool for detecting neurophysiological changes, and it assists in titrating anesthesia levels and prognosticating clinical outcomes, particularly in vulnerable patient populations [2^■^,^3^].

The significance of EEG in anesthesia lies in its ability to prevent intraoperative awareness and manage the risk of perioperative neurocognitive disorders by avoiding oversedation. EEG provides immediate feedback on brain activity, allowing anesthesiologists to adjust anesthetic dosages accordingly and avoid both overly light and excessively deep anesthesia [4]. However, despite its benefits, integrating EEG into standard practice faces challenges, including variability in EEG interpretation, high costs of monitoring devices, and a less defined role in pediatric populations [5^■^].

To facilitate the effective use of EEG in anesthesia, some training programs have been established to equip anesthesiologists with the skills to interpret EEG data accurately [6]. These educational initiatives emphasize a structured curriculum that includes theoretical and practical components, ensuring practitioners can recognize and respond to EEG changes resulting from different anesthetic agents. However, as EEG monitoring continues to evolve, its successful integration into anesthesia practice will depend on improved training methodologies, interdisciplinary collaboration, and ongoing research to refine its applications. This review seeks to examine current educational methods, highlight gaps in EEG training, and explore potential future improvements.

## CHALLENGES TO ELECTROENCEPHALOGRAPHY ADOPTION

Despite the physiological rationale and growing clinical evidence supporting EEG monitoring during anesthesia, its routine adoption remains limited, and multiple barriers contribute to this underutilization. A fundamental obstacle is that most anesthesiologists have not been adequately trained to interpret raw EEG signals. Historically, EEG interpretation was perceived as a specialized skill restricted to neurologists. Consequently, EEG training has been minimal or absent from standard anesthesiology curricula [7,8]. Recent studies demonstrated that even a short, structured educational session significantly improves anesthesiologists’ ability to interpret EEG waveforms [7,8]. This suggests the primary issue is the lack of opportunity for structured, accessible learning.

In addition, compared with the regular, rhythmic patterns of ECGs, EEG waveforms appear highly stochastic and irregular [8]. This perception of complexity can intimidate clinicians and discourage engagement. However, evidence shows that under general anesthesia, particularly with propofol or volatile anesthetics, EEG patterns become highly stereotypical and recognizable [9,10]. Slow-delta oscillations, frontal alpha spindles, and burst suppression are reproducible signatures that can be learned with appropriate education. While EEG complexity is real in the awake state, essential patterns are often consistent and straightforward enough for nonspecialists to recognize for intraoperative monitoring [11^■^].

Furthermore, many anesthesiologists have been taught to rely on processed EEG (pEEG) monitors that reduce complex EEG data into a single index, including the Bispectral Index (BIS, Covidien, Ireland), the Patient State Index (PSI) (Masimo, Irvine, California, USA), and Entropy (GE HealthCare, Chicago, Illinois, USA) [12]. Nonetheless, studies have shown that pEEG indices can be misleading, particularly under conditions such as neuromuscular blockade, where muscle artifact reduction falsely lowers the index, suggesting deeper anesthesia than is truly present [13]. Additionally, processed indices often do not account for agent-specific EEG changes or patient factors like age, leading to potential errors in clinical decision-making [14,15]. As such, clinicians who rely solely on indices without cross-referencing the raw EEG risk making anesthetic management decisions based on inaccurate interpretations.

EEG education still lacks standardized curricula within anesthesiology training programs. Until recently, EEG interpretation was not included in competency-based milestones for anesthesiology residents, leaving a significant gap in training [6]. Programs that attempt to introduce EEG often vary widely in content, depth, and rigor, making it difficult to achieve consistent competency across institutions. The developing a real-time electroencephalogram-guided anesthesia management curriculum for educating residents (DREAMER) trial, among others, highlights how structured, spectrogram-based EEG education can significantly improve knowledge and potentially impact patient outcomes, yet few residency programs have formally adopted such interventions [6].

Finally, there is a broader cultural resistance to adopting new monitoring modalities. Many clinicians are reluctant to introduce what they perceive as additional complexity or workload into an already busy operating environment [16]. Additionally, hospital systems may hesitate to invest in EEG-capable monitors or prioritize faculty development in EEG interpretation.

Changing these attitudes requires education and demonstrating that EEG monitoring can enhance patient safety, reduce anesthetic consumption, and potentially improve outcomes such as postoperative cognitive function [17^■^,^18^,19^■■^] and lead to a more sustainable anesthesia practice.

## CURRENT EDUCATIONAL APPROACHES

As EEG monitoring becomes more prevalent in daily clinical practice, training programs are emerging, enabling anesthesiologists to interpret EEG at the bedside [20]. The essential principles of EEG are becoming increasingly incorporated into the education of anesthesiology fellows, helping them understand its applications for assessing the depth of anesthesia and recognizing brain states under various anesthetic agents [21]. These initiatives aim to bridge the knowledge gap and ensure practitioners can effectively use EEG to monitor neurophysiological changes during anesthesia and sedation. Currently, EEG education programs employ a variety of teaching methodologies, including large-group lectures, small-group sessions, self-directed learning opportunities, and practical hands-on courses. Personalized learning strategies, content, and resources can be formulated by analyzing and studying different learning strategies [22].

## TEACHING METHODOLOGIES USED ACROSS PUBLISHED RESEARCH

Various educational methodologies have been used across reviewed studies to teach anesthesiologists how to interpret EEG during anesthesia. However, it must be emphasized that research on EEG education in anesthesiology remains extremely sparse. Only a few interventional studies and educational frameworks have been published, highlighting a major gap in the field and the need for further research and curriculum development. Table 1 summarizes diverse educational approaches used to teach EEG interpretation across neurology and anesthesiology training programs.

**Table 1.** Educational strategies for EEG interpretation: a comparative overview of teaching modalities across clinical learner levels

EEG learning modality	Example study	Key methods	Target audience	Outcomes
Video-based flipped classroom	Moeller *et al*. (2017) [23]	Short modular videos (up to 17 min), self-paced learning, embedded quizzes, used during rotation	Neurology residents	High engagement and satisfaction; perceived improvement in understanding; no objective performance data
Multidisciplinary module with deliberate practice	Fahy *et al*. (2019) [24]	Spaced learning, weekly sessions, active retrieval, clinical immersion, and direct EEG interpretation with feedback	Anesthesiology residents	Significant improvement in ITE scores; sustained long-term knowledge retention
Virtual EEG electrode placement simulator	Björn *et al*. (2020) [25]	PC-based 3D simulator with fuzzy/exact feedback systems, used in neurophysiology course	Biomedical science students	Improved theoretical knowledge and electrode placement skills compared to control group
Traditional hands-on EEG training	D’Onofrio *et al*. (2025) [26]	EEG lab rotations (3–6 months), supervised interpretation of real EEGs	Neurology residents/fellows (global)	Highly variable exposure; many trainees report inadequate confidence and competence
Synchronous/asynchronous online courses	Fernandez *et al*. (2023) [27]	Online modules, live sessions, assessments	Neurology residents	Improved postcourse test scores; effective supplemental format
Brief structured EEG training session	Bombardieri *et al*. (2020) [7]	35-minute didactic on EEG waveform recognition for hypnotic depth, pre/post-tests	Anesthesiologists (mixed experience levels)	Significant improvement in EEG interpretation for untrained clinicians; no improvement for previously trained
Didactic EEG training with testing	Barnard *et al*. (2007) [8]	15-min PowerPoint, EEG ranking tasks, pattern recognition training	Anesthesiologists (mixed experience levels) (New Zealand)	Most participants could correctly distinguish awake vs anesthetized EEG; performed similarly to BIS/entropy monitors
Continuing professional development module (CPD)	Hight *et al*. (2020) [10]	Structured CPD content on interpreting raw EEG and spectrogram in GA	Anesthesiologists (mixed experience levels)	Improved understanding of EEG-spectral features, limitations of pEEG indices highlighted
Advanced raw and processed EEG interpretation training	Lee *et al*. (2021) [16]	Review-based instruction on waveform, alpha power, spectrogram, and burst suppression; interpretation applied to clinical decisions	Anesthesiologists (advanced learners)	Suggested utility for titration and care in vulnerable populations; evidence-based support for pEEG-guided approaches
Educational Reviews with Illustrative Case Scenarios	Kim *et al*. (2020) [9]	Tutorial-style review with EEG-spectrogram interpretation across anesthetic drugs	Anesthesiologists (advanced learners) and educators	Promotes transition from index-based to raw EEG/spectrogram-based monitoring for personalized anesthesia

BIS, Bispectral Index; EEG, electroencephalography; GA, general anesthesia, ITE, the in-training examination; pEEG, processed EEG.

### Short, focused educational sessions

Bombardieri *et al*. [7] implemented a structured session to teach anesthesia clinicians to identify raw EEG waveforms associated with different hypnotic depths, including wakefulness, nonslow-wave anesthesia, slow-wave anesthesia, and burst suppression. The teaching used simple explanations and abundant visual examples to illustrate key EEG patterns. Pre- and posteducational tests allowed participants to engage with the learning and assess their understanding and improvement. Similarly, Barnard *et al*. [8] conducted a short lecture emphasizing the key differences between the EEG patterns seen during wakefulness and those observed under anesthesia. The session focused heavily on pattern recognition, using numerous visual examples to aid learning. Participants were immediately tested on their ability to differentiate between awake and anesthetized EEG states.

Finally, the study by Moeller *et al*. [23] evaluated a video-based EEG curriculum designed to teach foundational electroencephalography concepts to neurology residents across two academic centers. The curriculum consisted of short modular videos covering essential topics such as EEG terminology, regular patterns, artifacts, and basic abnormalities. The program allowed residents to engage with content independently before or during their EEG rotations, enhancing the efficiency of faculty-led teaching. Survey results showed high engagement and satisfaction: 87% of residents watched at least half the videos. Though objective performance data were not collected, residents perceived the curriculum as an effective and flexible tool for improving their understanding of EEG interpretation. Together, these studies strongly suggest that even very brief, targeted educational interventions can meaningfully improve EEG interpretation skills among anesthesiologists.

### Electronic-learning curriculum

In the DREAMER Trial [6], the authors developed an electronic-learning curriculum combined with bedside reinforcement to teach residents about EEG-spectrogram interpretation and its application to anesthetic titration. The electronic modules included quizzes and interactive elements to engage learners actively and align with Millennials and Generation Z preferences [28]. After completion, trainees had real clinical exposure by using spectrogram monitors intraoperatively to practice their skills. This strategy encouraged active application of theoretical knowledge in the clinical environment immediately after training.

### Real-time clinical practice with feedback

Also in the DREAMER Trial [6], residents were encouraged to use EEG spectrograms during live surgeries, with faculty members providing guidance and feedback. This direct application of knowledge allowed residents to consolidate their skills through real-world clinical immersion.

### Deliberate practice

The study by Fahy *et al*. [24] demonstrates an educational approach to teaching EEG interpretation that aligns with cognitive science principles, including spaced learning [29], active retrieval (effortful recall of information) [30], interdisciplinary clinical immersion [31], and multifaceted dynamic teaching (where the students are engaged and active participants in their education) [32]. Spaced learning was deliberately implemented by scheduling EEG interpretation sessions once a week. This approach leveraged the well-established ‘spacing effect’ [29], which shows that material learned over distributed sessions is retained significantly longer than material presented in a single session. The module incorporated active retrieval practice: [30] residents were assessed multiple times – at the beginning of the rotation, immediately after didactic instruction, and again at its conclusion. The program also embedded the principles of deliberate practice [33,34]. During weekly 1-hour sessions with neurophysiologists, residents interpreted real EEG tracings, discussed patient-specific clinical correlations, and received structured, targeted feedback on their performance. This cycle of repetition, feedback, and incremental challenge allowed residents to refine their analytical skills in a focused and supportive learning environment, promoting progression toward true mastery of EEG interpretation. The effectiveness of this comprehensive approach was evident in objective outcomes. Residents who completed the EEG module scored significantly higher on EEG-related questions in the in-training examination (ITE) than their peers who did not receive the training and the national average, even though many residents took the ITE a year after completion of the program, indicating robust long-term knowledge retention [35].

### Use of simulation

Teaching EEG interpretation using simulation is increasingly supported by evidence demonstrating its effectiveness in accelerating skill acquisition, improving pattern recognition, and enhancing clinical decision-making [36,37,38^■^,^39^]. Studies have shown that simulation-based EEG training, whether through high-fidelity patient simulators or virtual platforms, allows learners to engage with dynamic EEG patterns in real-time, promoting active learning and immediate feedback [36,37,38^■^]. This method has been associated with better knowledge retention than traditional didactic teaching alone, particularly for complex concepts [40]. Moreover, simulation offers a safe environment for repeated practice without risk to patients [39]. Overall, integrating simulation into EEG education aligns with adult learning principles and competency-based education models, making it a powerful tool for developing proficiency in novice and advanced learners [26].

## CURRICULUM STRUCTURE

A well-defined curriculum is crucial for EEG education in anesthesiology. The curriculum should outline competencies expected from trainees, ensuring they engage actively with supervising faculty [41]. Berger-Estilita *et al*. [42] recently employed a Delphi consensus process to define a structured framework for EEG education in anesthesiology. The experts agreed that EEG education should follow a tiered approach, starting with basic waveform recognition and progressing toward complex topics such as patient variability, artifacts, and nociception. They recommended adopting a spiral curriculum model, where core concepts are revisited and deepened over time.

Additionally, they advocated for competency-based education, emphasizing the development of skills and clinical application rather than mere accumulation of theoretical knowledge. The consensus document supports structured learning paths progressing from basic to advanced levels, with repeated reinforcement of essential concepts. This competency-driven model ensures learners achieve proficiency through skill-based progression rather than relying solely on time-based metrics.

## SELF-DIRECTED LEARNING AND INDEPENDENT STUDY

In addition to structured curricula and simulation-based training, self-directed learning is critical in developing EEG interpretation skills among anesthesiologists. Self-study resources, such as online EEG video libraries [13,43], interactive e-learning platforms [43–45], and scientific publications [6–8,46–48], allow learners to engage with EEG education at their own pace and according to their individual learning needs. Self-directed learning enables repetitive exposure to EEG patterns, promotes active engagement through case-based exercises, and allows for immediate application of knowledge in clinical contexts [49]. Furthermore, when combined with periodic faculty feedback or peer discussion sessions, self-study can significantly reinforce understanding and address specific knowledge gaps. As EEG education advances, integrating high-quality, accessible self-learning tools will be essential to complement formal instruction, facilitate lifelong learning, and support mastery of this critical anesthetic monitoring modality.

**FIGURE 1.**
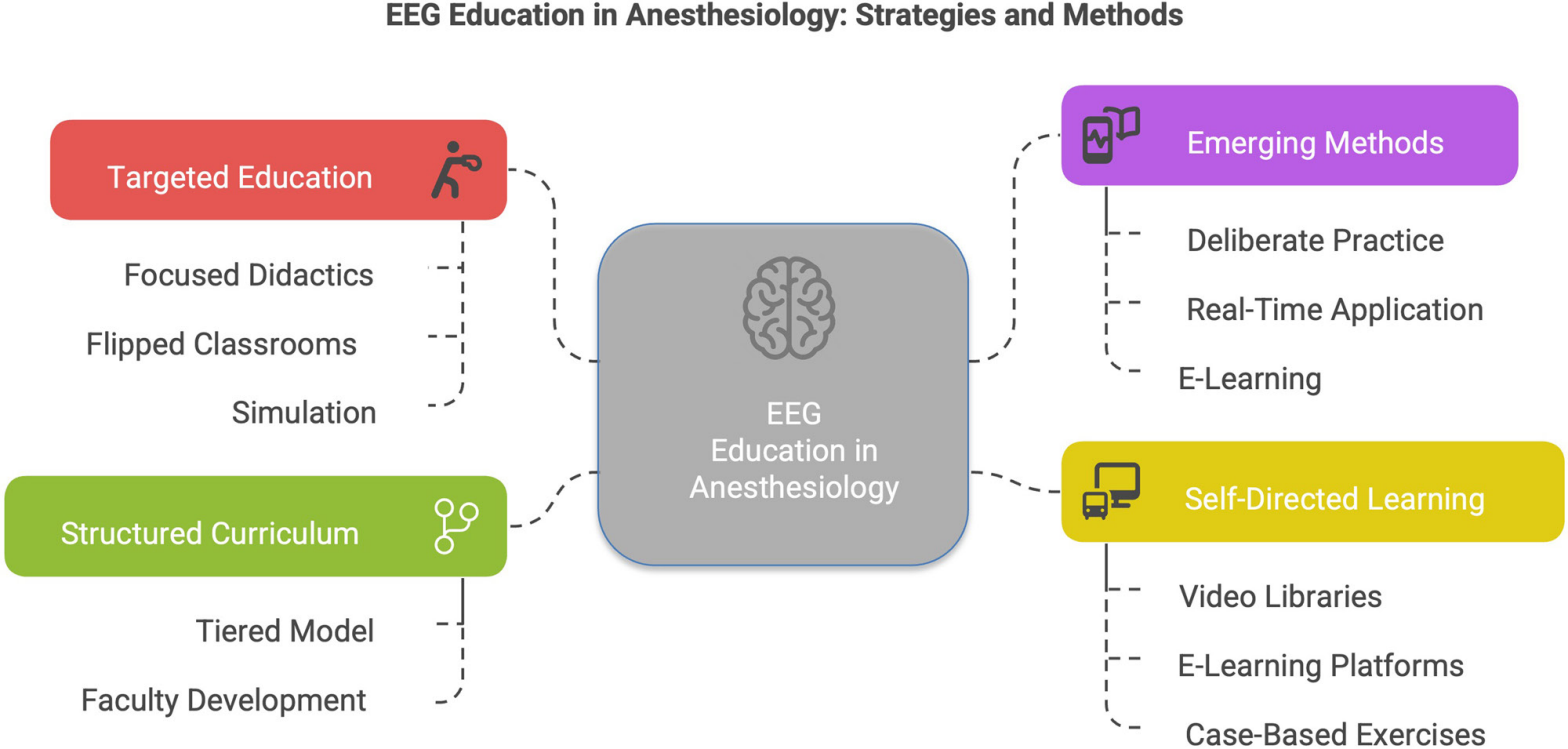
Electroencephalography (EEG) education in anesthesiology: strategies and methods.

Figure 1 provides a visual summary of the key teaching methodologies currently used in EEG education for anesthesiologists, consolidating the diverse educational approaches described above.

## FUTURE DIRECTIONS

As anesthesiology evolves in response to technological advancements and changing learner profiles, future directions in EEG education must align with the expectations and learning preferences of new generations of trainees. Millennials and Generation Z increasingly demand flexible, technology-enhanced, and purpose-driven education [28]. To meet these needs, anesthesia training will likely incorporate adaptive learning platforms that use artificial intelligence to personalize content delivery, modular microlearning formats for mobile access, and gamification strategies to boost engagement and retention. Augmented and virtual reality tools may offer immersive EEG simulations that replicate intraoperative scenarios with real-time feedback. Moreover, the trend toward interprofessional and interdisciplinary training environments – fostering collaboration between anesthesiologists, neurologists, and data scientists – will better equip trainees to handle the complexity of modern perioperative care. Future curricula must also prioritize continuous, lifelong learning, offering just-in-time EEG resources and maintenance of certification pathways that ensure sustained competency. Ultimately, adapting anesthesia education to the evolving digital landscape and generational learning styles will be essential to normalize EEG interpretation as a core clinical skill and to enhance perioperative brain health.

## CONCLUSION

Improving perioperative brain health includes practical, accessible, and clinically relevant EEG training. Programs should simplify interpretation using pattern-based teaching, highlighting recognizable EEG signatures. Training should emphasize integrating raw EEG into routine anesthetic care, encouraging validation of processed indices against the raw signal to avoid artifacts. Embedding EEG education into anesthesiology residency milestones and ongoing professional development will ensure lasting competency. Faculty training is also crucial to foster confident EEG use. These steps are key to making EEG a standard, patient-centered tool in anesthesia.

## Acknowledgements

This manuscript was initially conceptualized during online meetings. All authors have substantially contributed to conception and design, drafting the article or revising it critically for important intellectual content. All authors have read and approved the final manuscript; and agree to be accountable for all aspects of the work thereby ensuring that questions related to the accuracy or integrity of any part of the work are appropriately investigated and resolved.

## Financial support and sponsorship

None.

## Conflicts of interest

J.B.E. is a member of the Board of Directors of the European Society of Anaesthesiology and Intensive Care (ESAIC) and has received speaker’s fees from Medtronic. S.S. is the lead of ESAIC’s subcommittee for the Geriatric Patient and has received speaker’s fees from Medtronic/Merck.
